# User experience with the Ask Your Pharmacist teleconsultation platform

**DOI:** 10.1177/17151635211006446

**Published:** 2021-05-03

**Authors:** Line Guénette, Alexandre Chagnon, Véronique Turcotte

**Affiliations:** Axe Santé des populations et pratiques optimales en santé, Centre de recherche du CHU de Québec-Université Laval, Laval University, Quebec City; Faculty of Pharmacy, Laval University, Quebec City; Centre intégré universitaire de santé et de services sociaux de l’Estrie–Centre hospitalier universitaire de Sherbrooke, Granby, QC; Axe Santé des populations et pratiques optimales en santé, Centre de recherche du CHU de Québec-Université Laval, Laval University, Quebec City

## Abstract

**Background:**

An increasing number of pharmacists use technology and social media to connect with patients. However, such means may pose confidentiality issues and legal problems. To correct this situation, a platform of teleconsultation services provided by pharmacists, titled “Ask Your Pharmacist,” was created in Quebec, Canada.

**Methods:**

A web-based satisfaction survey was carried out among patients and pharmacists who have used the Ask Your Pharmacist platform to describe their experience and satisfaction with the platform and explore the perceived usefulness of this service in the province of Quebec.

**Results:**

A total of 53 patients and 27 pharmacists completed the survey. Most patients were satisfied or very satisfied with their experience with Ask Your Pharmacist (96.2%), said that it met their need (88.7%), and agreed they would not have to consult again about the matter discussed with the pharmacist (75.5%). The main motivation of pharmacists for volunteering on Ask Your Pharmacist was to meet the needs of patients (85.1%), promote their profession (55.6%), improve drug utilization in the population (55.6%) and increase accessibility to a pharmacist (51.9%). Most (81.5%) felt that providing written consultation (rather than oral) required more research on their part.

**Discussion:**

Most patients judged they would not have to have another consultation about the matter discussed with the pharmacist, suggesting that Ask Your Pharmacist may avoid the need for physician and emergency department visits.

**Conclusion:**

Most patients and pharmacists were satisfied with their experience with Ask Your Pharmacist and perceived this service as useful. Further studies should assess the impact of this platform on the utilization of other health care services. *Can Pharm J (Ott)* 2021;154:xx-xx.

**Knowledge Into Practice**

- There is an increasing demand from patients to interact electronically with care providers, and therefore, an increasing number of pharmacists use technology and social media to connect with patients.
- Most patients and pharmacists were satisfied with their experience of an asynchronous teleconsultation platform provided by pharmacists and perceived this service as useful.
- The asynchronous format of the Ask Your Pharmacist platform might facilitate the integration of this type of service in pharmacists’ busy practice.
- The Ask Your Pharmacist teleconsultation platform appeared to improve awareness about pharmacy services and pharmacists’ expertise among the users.

**Mise En Pratique Des Connaissances**

- Les patients demandent de plus en plus à interagir électroniquement avec les prestataires de soins et, par conséquent, un nombre croissant de pharmaciens utilisent la technologie et les médias sociaux pour entrer en contact avec les patients.
- La plupart des patients et des pharmaciens étaient satisfaits de leur expérience d’une plateforme de téléconsultation asynchrone fournie par les pharmaciens et ont perçu ce service comme utile.
- Le format asynchrone de la plateforme Question pour un pharmacien pourrait faciliter l’intégration de ce type de services dans la pratique active des pharmaciens.
- La plateforme de téléconsultation Question pour un pharmacien a semblé améliorer la sensibilisation des utilisateurs aux services pharmaceutiques et à l’expertise des pharmaciens.

## Introduction

The general population is increasingly using information and communication technologies to seek therapeutic information. In 2012, 59% of Americans had used the Internet during the year to find health information.^1^ A study conducted for the Canadian Radio-television and Telecommunications Commission (CRTC) also showed that 90% of Canadians accessed the Internet “at least occasionally” to search for medical or health-related information in 2016, while this proportion was only 64% in 2010.^2^ According to Statistics Canada, the type of information sought by users varies by age and sex. Among people aged 18 to 44 years, 34% of men compared with 41% of women used the Internet to access information on drugs. These proportions are 44% and 48% among men and women aged 45 years and older, respectively.^3^

In addition, an increasing number of pharmacists use technology to connect with patients and to provide telepharmacy services. As noted by the CEFRIO in 2016, more than one-third of Quebec-based community pharmacists were using Facebook to “provide advice” to patients (36%) and to promote their services (35%).^4^ Using such a platform for patient communication may expose both patients and pharmacists to confidentiality and legal issues.

Ask Your Pharmacist is a teleconsultation platform (https://askyourpharmacist.ca) that aims to correct this situation. It is a consumer-facing website, pooling the availability and expertise of more than 500 pharmacists in the province of Quebec, Canada. These volunteer pharmacists offer a consultation service to better inform the public about the appropriate use and expected impacts of drugs and also to connect with nearby information-seeking patients. Anyone aged 14 years and older can ask a question. The question is sent simultaneously to several pharmacies registered on the Ask Your Pharmacist platform. Those pharmacies are selected depending on their geographical location, with a preference to pharmacies nearest to the person asking the question. When a pharmacist writes the answer on the platform, the person receives an email notification that prompts the patient to return on the platform to read the answer. If necessary, a discussion in text messaging format (short message service)—a chat—can occur between the pharmacist and the patient. Every question is answered within a 24-hour period by one of the pharmacists. If she or he wishes to, the responding pharmacist can then publish its answer, which will be available for browsing by other patients, with more than 12,500 other answers. This strategy allows pharmacists to reach more patients with a unique answer. This service has been offered free of charge since October 2015 (and in its current format since March 2019) and has provided information to more than 50,000 and 120,000 Quebecers in 2019 and 2020, respectively.

Pharmacists involved on the platform are interested in acquiring a new clientele by being connected with patients living near their pharmacy. Thus, the benefit of volunteering comes from the potential to get new patients into the pharmacy. Pharmacists are responsible for answering patients’ questions, the same way they do when they answer questions over the phone. Their professional insurance covers their activities on the Ask Your Pharmacist platform. The platform is managed by a company owned by Quebec-based pharmacists and funded by displaying ads to users. These ads are shown to users based on their interests. For instance, patients asking questions about a newborn could see ads related to infant formula. To ensure that every encounter between patients and pharmacists is secure and confidential on the Ask Your Pharmacist platform, all personal health information is hosted in Canada. Notification emails are sent to users to let them know of a new interaction with their question or answer. Moreover, the platform managers ensure all questions and answers are anonymized prior to publication on Ask Your Pharmacist.

The objectives of this study were to evaluate the Ask Your Pharmacist platform by describing the experience and appreciation of users (patients and pharmacists) and by exploring the perceived usefulness of this service in the province of Quebec.

## Methods

### Study design and population

We conducted a cross-sectional study in 3 phases (Summer, Fall and Winter) between July and December 2018. An email invitation to participate in a web-based satisfaction survey was sent to all patients who had asked a question on the Ask Your Pharmacist platform and to all pharmacists who had provided at least 1 consultation during these periods. This corresponded to 247 patients and 38 pharmacists. A reminder email was sent 2 weeks after the first email. We used Questback EFS Survey Software version 10.9 to build the online questionnaire.

### Data collection and variables

The online patient survey included 20 questions, while the pharmacists’ survey had 15 questions. The questionnaires included questions covering sociodemographic characteristics (e.g., sex, region of residence or practice) and their experience with Ask Your Pharmacist (e.g., number of questions asked or answered, their satisfaction with the information provided). Most of the questions were closed ended, with multiple-answer choices or 4-point Likert-type scales. On certain occasions, an “other” option was provided to allow the participants to enter free-text answers if their preferred answer was not listed. The questionnaire was available in French since it is the language most frequently spoken by users (77.1% of the population in the province of Quebec speaks French). The Research Ethics Board of the CHU de Québec-Université Laval Research Center approved this study (No. 2019-4220).

### Analysis

A descriptive analysis was performed. The results were discussed between researchers and with the Ask Your Pharmacist platform development team.

## Results

### Characteristics of patients and pharmacists participating in the survey

A total of 53 patients and 27 pharmacists completed the survey, which corresponds to participation rates of 21.5% and 71.1%, respectively. The characteristics of the patients are presented in Table 1. They were mainly women (83%), aged 45 to 54 years (30.2%), had a college degree as their highest level of education (28.3%) and were located in different regions of the province of Quebec. Some of them (7.5%) reported having no general practitioner. Table 2 presents the characteristics of the pharmacists. Most of them were women (55.6%), had more than 10 years of experience as a pharmacist (40.7%) and were practising in community pharmacies (96.3%) in different administrative regions of the province. Two regions had no participants, either patients or pharmacists: Bas-St-Laurent and Nord-du-Québec.

**Table 1:** Characteristics of patients participating in the survey

	Patients	
Characteristic	*N* = 53	%
Sex		
Women	44	83.0
Men	9	17.0
Age, years		
18-24	14	26.4
25-34	4	7.5
35-44	6	11.3
45-54	16	30.2
55-64	9	17.0
65-74	4	7.5
Highest degree of schooling		
None or primary school	3	5.7
Secondary school diploma	7	13.2
Vocational school diploma	7	13.2
College degree	15	28.3
University certificate	7	13.2
Bachelor’s degree	7	13.2
Master’s degree	7	13.2
Region of residence		
Abitibi-Témiscamingue	1	1.9
Bas-St-Laurent	0	0
Capitale-Nationale	7	13.2
Centre-du-Québec	6	11.3
Chaudières-Appalaches	1	1.9
Côte-Nord	0	0
Estrie	7	13.2
Gaspésie-Iles-de-la-Madeleine	1	1.9
Lanaudière	2	3.8
Laurentides	3	5.7
Laval	2	3.8
Mauricie	2	3.8
Montérégie	9	17.0
Montréal	6	11.3
Nord-du-Québec	0	0
Outaouais	4	7.5
Saguenay-Lac-St-Jean	2	3.8
Have a general practitioner		
Yes	49	92.5
No	4	7.5
Type of device used to access Ask Your Pharmacists*		
Computer	34	64.2
Tablet (e.g., iPad)	6	11.3
Smartphone	21	39.6

* Total exceeds 100% as more than 1 answer was possible.

**Table 2:** Characteristics of pharmacists participating in the survey

	Pharmacists	
Characteristic	*N* = 27	%
Sex		
Women	15	55.6
Men	12	44.4
Number of years of experience as a pharmacist		
0-2	7	25.9
3-5	4	14.8
6-10	4	14.8
> 10	11	40.7
Missing	1	3.7
Type of pharmacy practice*		
Community pharmacy	26	96.3
Health institution	2	7.4
Family medicine group	2	7.4
Other	1	3.7
Region of practice		
Abitibi-Témiscamingue	0	0
Bas-St-Laurent	0	0
Capitale-Nationale	4	14.8
Centre-du-Québec	0	0
Chaudières-Appalaches	2	7.4
Côte-Nord	1	3.7
Estrie	1	3.7
Gaspésie-Iles-de-la-Madeleine	0	0
Lanaudière	4	14.8
Laurentides	6	22.2
Laval	0	0
Mauricie	0	0
Montérégie	3	11.1
Montréal	3	11.1
Nord-du-Québec	0	0
Outaouais	2	7.4
Saguenay-Lac-St-Jean	1	3.7
Number of months of involvement on Ask Your Pharmacist		
≤5	4	14.8
6-12	13	48.1
13-20	2	7.4
>20	8	29.6
Number of questions answered on Ask Your Pharmacist		
1-3	7	25.9
4-10	7	25.9
11-20	7	25.9
21-30	1	3.7
>30	3	11.1
Do not know	2	7.4
Type of device used to access Ask Your Pharmacists*		
Personal computer	23	85.2
Personal tablet (e.g., iPad)	3	11.1
Personal smartphone	15	55.6
Pharmacy computer	6	22.2

* Total exceeds 100%, as more than 1 answer was possible.

### Experience of patients with Ask Your Pharmacist

Most of the 53 patient-users (88.7%) reported asking the question for themselves and 60.4% had previously consulted the available answers on the platform. Thirty-two patient-users (60.4%) mentioned it was the first time they had asked a question. Twenty-one (39.6%) patients had already discussed the matter with a health care professional, either a general practitioner (11/21), a pharmacist (9/21) or another health care professional (6/21). Most patient-users were aware of Ask Your Pharmacist because of their Internet search (45.3%) or through social media (18.9%). Very few had heard of this service through a health care professional (9.4%).

Most patients were satisfied with their experience with Ask Your Pharmacist. In fact, 96.2% reported being satisfied or very satisfied with the response provided by the pharmacist, while 88.7% said that it answered their need (moderately to completely). Virtually all participants (96.2%) said they did not have to visit the pharmacy to meet the pharmacist in person to complete the consultation. All participants said the answer was easy or very easy to understand, and almost all of them (94.3%) thought that the delay for obtaining a response was reasonable. Very few participants (5.7%) thought that the answer did not contain enough information. The mean probability that they would recommend Ask Your Pharmacist to a friend or a family member was also very high (95.5%).

### Perceived impacts of using Ask Your Pharmacist

**Figure 1:**
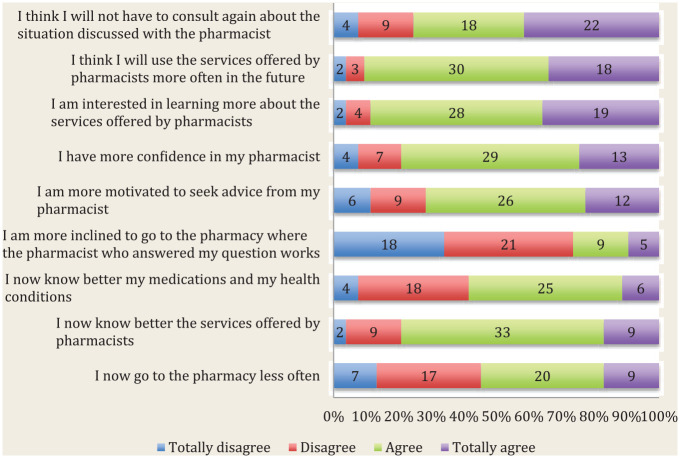
Patient-users’ perceived impact of using the Ask Your Pharmacist teleconsultation platform

The impacts of using Ask Your Pharmacist from the patient-users’ perspective are presented in Figure 1. This service seems to be effective, as most patients participating (75.5%) agreed they would not have to consult again about the matter discussed with the pharmacist. Ask Your Pharmacist appears to be a service improving awareness about pharmacy services and pharmacists’ expertise, with more than 70% of patients agreeing with these statements. Also, Ask Your Pharmacist does not seem to replace the services provided by pharmacists practising in traditional community pharmacies, with only 54.7% of participants agreeing they now go less often to the pharmacy.

### Experience of pharmacists with Ask Your Pharmacist

The 27 pharmacists completing the survey reflected these last perceptions by patients. Indeed, most (88.9%) reported that none of the patients visiting their pharmacy had ever told them they had used their services on the Internet. Their main motivation for volunteering on Ask Your Pharmacist was to meet the needs of the patients (85.1%), promote the profession (55.6%), improve drug utilization in the population (55.6%) and increase accessibility to a pharmacist (51.9%).

As for their experience with Ask Your Pharmacist, most pharmacists (77.8%) answered questions from their home always or most of the time. Most pharmacists (74.1%) thought it took them between 6 and 15 minutes to answer a question. The vast majority (85.2%) found that the questions they answered through Ask Your Pharmacist were easy or very easy to understand, but most (51.9%) thought that patients did not provide enough information in their question. They also perceived that the fact that their consultation was written instead of oral required more research (81.5%) from their part. Moreover, 55.6% reported that they always or most of the time referred to evidence-based information sources.

## Discussion

Most patients were satisfied with their experience with Ask Your Pharmacist. They thought this service answered their need and was effective even with the asynchronous format. This level of satisfaction was also observed in a study by Ho et al.^5^ about a chat-based telepharmacy service in Denmark and for a teleconsultation service provided to HIV outpatients in Spain.^6^ Ask Your Pharmacist also appeared to improve awareness about pharmacy services and pharmacists’ expertise. Those results are in line with a previous study published in 2018 by Chagnon and Vandesrasier.^7^ As for the pharmacists’ experience, the asynchronous format certainly facilitates their use of the Ask Your Pharmacist platform. Indeed, with this format, pharmacists do not need to offer appointments and may answer questions when they are free. This feature of Ask Your Pharmacist might be particularly important, as pharmacists are not paid for this service and most of them answered questions from their home. Moreover, it allows them to take their time to provide complete answers and to cross-check with evidence-based information sources if needed.

It is surprising that a large proportion of pharmacists mentioned that none of the patients visiting their pharmacy had ever told them they had used their services on the Internet. This could be due to the fact that those pharmacists do not have the time to ask new patients such a question. A study conducted in the United States showed that an asynchronous telehealth service held by doctors achieved a conversion rate (defined as the proportion of patients who became regular clients of their clinic after completing an e-visit with a physician with whom they had never interacted) of 24.8%.^8^ This study represents the bedrock on which 2 platforms similar to Ask Your Pharmacist were created: HealthTap (https://www.healthtap.com) in the United States and Healthshare (https://www.healthsharedigital.com) in Australia. Telepharmacy services might also be different in terms of conversion rates than other telehealth services. To allow pharmacists to estimate how many patients were acquired from the Ask Your Pharmacist platform, an email survey is now sent to all individuals who asked a question 7 days following their interaction with the pharmacist. This survey collects information about whether they visited the pharmacy in the hours that followed receipt of the pharmacist’s answer and about their appreciation of the service provided. This information is then provided to pharmacists on a personal dashboard with the aim to improve their practice.

Patient demographics are aligned with the fact that a rising number of older adults (age 65 years and older) are online every day.^9^ Those metrics also show the importance of pharmacists investing in the digital space and meeting patients where they are and where they need help the most. Most patients participating in the current study said they would not have to consult again about the matter discussed with the pharmacist, suggesting that using Ask Your Pharmacist may allow patients to avoid medical clinics and emergency department consultations. Similarly, at a medical clinic in the United Kingdom, the delivery of a telephone consultation with a pharmacist led to a 50% reduction in the number of patients requiring a same-day face-to-face appointment with their general practitioner.^10^ As well, a study performed in Ontario found that almost one-third of all avoidable emergency department visits may potentially be manageable by pharmacists within their expanded scope of practice as authorized in Canadian jurisdictions. This represents 4.3% of all emergency department visits.^11^ Thanks to the present study, a pilot project with the provincial ministry of health was launched in September 2020. The project prompts nurses of the 8-1-1 telephone consultation service to redirect questions related to medication to community pharmacists. Pharmacists will be remunerated, as our study suggests that this service is valuable and could reduce the need for other consultations.

Since the creation of the Ask Your Pharmacist platform in 2015, a few other teleconsultation services offered by pharmacists have been published online. To our knowledge, patients from the United Kingdom (https://askhala.com), Africa (http://www.santeclic.com/questions/), France (http://www.mapharmaciemobile.com/article/les-services-a2.html#conseil), the United States (https://www.walgreens.com/rx-utility/pharmacychat) and Denmark (https://www.apoteket.dk/raadgivning/chat#) can also now send their questions online to connected pharmacists. However, Ask Your Pharmacist is currently the only asynchronous telehealth service involving pharmacists. With the current COVID-19 pandemic and the increasing demand from patients to interact electronically with their care providers, such initiatives and services will certainly increase over the next years. Despite this growing interest, this is one of the first studies assessing a telepharmacy service for online remote consultations offered to patients.^12^

This study has several strengths. First, the survey was sent to patients and pharmacists soon after the patient received the pharmacist’s answer. Second, the survey was sent at 3 different time points to avoid a seasonal bias and to capture various experiences. Some limitations must also be acknowledged. The answers shared regarding the Ask Your Pharmacist platform might not be generalizable to all patient-users and registered pharmacists as only a limited number of them answered the survey. Some characteristics of the patients (e.g., their young age) and the pharmacists (e.g., their experience) may have influenced the results. Moreover, no response was received from patients of 3 regions of Quebec province: Bas-Saint-Laurent, Côte-Nord and Nord-du-Québec. However, at the time of writing this article, only 1% of all questions received on the platform were asked by patients living in one of these sparsely populated regions. As for pharmacists, only those having a recent experience in answering a question were solicited (*n* = 38), but we reached an appreciable participation rate (71.1%). We believe that this restriction to recent users was necessary considering we were interested in their experience and satisfaction with the Ask Your Pharmacist platform and that many registered pharmacists have never answered a question. We also have to acknowledge that the usefulness and impact of the platform is based on the participants’ perception.

## Conclusion

Most patients and pharmacists were satisfied with their experience with Ask Your Pharmacist and perceived this service to be useful. Patients thought this service answered their need and was effective and also appeared to improve awareness about pharmacy services and pharmacists’ expertise. Further studies should assess the impact of the platform on the utilization of care and services, as this evaluation was based on perceptions.
